# The “School of the Job”

**Published:** 1912-07-15

**Authors:** Wilbur F. Litch


### THE "SCHOOL OF THE JOB"

BY WILBUR F. LITCH, D.D.S.

Broadly stated education has a threefold purpose: First, the training of the moral nature and the physical organism; second, the training of the intellectual powers through the communication of general knowledge; and third, the specific training of all the varied powers, moral, mental and physical, needed for the successful prosecution of some selected occupation, through which the means of individual life may be secured and the general co-operative social life be preserved and perpetuated.

It being thus evident that the ultimate end of a man's education is to fit him to do a man's work, it is manifest that the kind of work he is to undertake should at as early a period as possible be definitely determined, in order that the general trend of his training may be made to the greatest possible extent contributory to his vocational success and usefulness.

The first and most important requisite for any high degree of success in any vocation is fitness, physical, mental and tempermental; for the best possible training is powerless to get good results from unfit material. Hence in the interests of the prospective worker character and vocational adaptability should be made the subject of careful observation throughout his educational career.

It is obvious that the boy without mechanical skill or liking for mechanics will make a failure as a handicraftsman. A poor arithmetician will not succeed as an accountant. Indifference to gain and lack of shrewdness in a dicker do not indicate the successful tradesman in embryo. Of the professions each demands some special quality or qualities without which success in its practice cannot be hoped for.

Vocational misfits not only entail an economical loss because they fail to get out of a man the best there is in him, but so inevitably result in personal unhappiness that they must be counted as among the chief of life's minor tragedies, and as not infrequently the source of some of the most bitter and poignant experiences in human existence. That man is indeed blessed who has found his work and rejoices in it because it is good.

Although so vital in its influence upon success or failure, happiness or unhappiness in life, the selection of a vocation is too often decided haphazard, without intelligent consideration of personal fitness or even of the immediate or ultimate economic requirements and possibilities of the chosen calling. This is markedly true of the professions and lamentably true of medicine and surgery, whether general or specialized.

No one who has had much experience as a teacher in a dental school can have failed to observe numerous instances in which the choice of dentistry as a profession has been most unwisely made, either because of inaptitude for mechanics, inability to grasp, retain and apply scientific principles, want of means, or lack of the physical stamina and the inherent magnetic force essential to gain and hold a practice. Fortunately many who for one ill-considered reason or another have decided to study dentistry discover their unfitness early in their novitiate as students and, recognizing their mistake, are wise enough to retire. Thus there is a sifting process which in the first year course of dental schools usually creates a considerable discrepancy between the number originally registered and the number in actual attendance at the end of the term.

Many who remain have good reason to regret that they had not been better advised. One such—an impoverished dental student in a Chicago school—but a few weeks ago, hard pressed for means, became a suicide, leaving clutched in his hand this unfinished letter, a pathetic memorial of disillusion, discouragement and despair: "Dear, dear angel mother, I never could understand why I left the old farm and came here to study a profession. I was happy when I was with you on the little farm and teaching school in that little red schoolhouse down the road. But here in this sickening city——"

College faculties are often unjustly blamed for admitting disqualified students; but it must be remembered that the student material comes to them in a lump and that it takes time to sort it over and much more time to fully test its quality and fitness. When the educational value of manual training is so fully recognized that the training of eye and hand in constructive work will be made a part of the education of every school boy, each boy's natural mechanical aptitude can be ascertained with some degree of definiteness, thus placing at the command of the dental teacher a valuable source of information as to the probable capacity of prospective students for the delicate technical work required of the dental operator. Their mental capacity for the theoretical studies must be assumed if they have met the present entrance requirement of a four year's high school course, or its equivalent, as established by a strict examination by a competent and unbiased school official.

While there are no possible safeguards by which the tragedy of vocational mistake and failure can be entirely prevented, society owes it to itself to see that the possibility of such mistakes shall be minimized. The aspirant for a business or professional career should know with some degree of accuracy the nature of and conditions attending the undertaking, and this before and not after he has definitely committed himself to its pursuit as a life work. Hence such information must be given as an important feature of his preliminary education in school or academy.

In Luneburg, Germany, the superintendent of the gymnasium has sought to impart to his students this much needed information by lectures delivered by representatives of various pursuits, professional, business and technical, in which their respective requirements, advantages and disadvantages, the personal qualifications demanded and the cost of preparation are fully explained. At these lectures the parents of students are invited to be present, ask questions, and join in discussion. Because of information thus obtained it is stated that many students have entirely changed their plans as to the selection of their life work.

In New York, where 21,000 children leave school every year to earn a living, a Student's Aid Committee of the High School Teacher's Association is actively engaged in trying to place students after leaving school in positions for which they are best fitted. This has been tersely termed the "Graduate School of the Job."

The view that vocational guidance should form a part of school instruction is rapidly gaining ground and doubtless will soon crystallize into action by the establishment of vocational bureaus, the duty of which shall be to study the pupil and give to him and to his parents advice as to the choice of an occupation.

The plan appears to be entirely practicable. While it would not be wise to add to a course of instruction already burdened with detail beyond the capacity of the average student to master with any real thoroughness, information regarding the choice of a life pursuit is so much more important than a smattering of ologies and foreign tongues, with mastery of none, that for some one of such studies a short course on the selection of a vocation could be advantageously substituted. There should be an Undergraduate, as well as a Graduate, "School of the Job."—*The Dental Brief.*

